# TM1-IR680 peptide for assessment of surgical margin and lymph node metastasis in murine orthotopic model of oral cancer

**DOI:** 10.1038/srep36726

**Published:** 2016-11-09

**Authors:** Annie A. Suganya S., K. J. Kochurani, Madhumathy G. Nair, Jiss Maria Louis, Santhosh Sankaran, R. Rajagopal, K. Santhosh Kumar, Parvin Abraham, Balagopal P. G., Paul Sebastian, Thara Somananthan, Tessy Thomas Maliekal

**Affiliations:** 1 Cancer Research Program, Rajiv Gandhi Centre for Biotechnology, Thiruvananthapuram, Kerala, 695014, India; 2 Animal Research Facility, Rajiv Gandhi Centre for Biotechnology, Thiruvananthapuram, Kerala, 695014, India; 3 Chemical Biology Group, Rajiv Gandhi Centre for Biotechnology, Thiruvananthapuram, Kerala, 695014, India; 4 Surgical Oncology, Regional Cancer Centre, Thiruvananthapuram, Kerala, 695011, India; 5 Division of Pathology, Regional Cancer Centre, Thiruvananthapuram, Kerala, 695011, India

## Abstract

Treatment outcome after surgical removal in oral carcinoma is poor due to inadequate methodologies available for marking surgical margins. Even though some methodologies for intraoperative margin assessment are under clinical and preclinical trials for other solid tumours, a promising modality for oral cancer surgery is not developed. Fluorescent-based optical imaging using Near Infrared (NIR) dyes tagged to tumour specific target will be an optimal tool for this purpose. One such target, Gastrin Releasing Peptide Receptor (GRPR) was selected for the study, and its binding peptide, TM1-IR680, was tested for its efficacy for surgical margin prediction in murine orthotopic model of oral cancer, derived from primary samples. Here, for the first time in a preclinical analysis, we show that the size and margin of oral cancer can be predicted, as revealed by 3D-imaging. Interestingly, the peptide was sensitive enough to detect lymph nodes that harboured dispersed tumour cells before colonization, which was impossible to identify by conventional histopathology. We recommend the use of TM1-NIR dyes alone or in combination with other technologies to improve the clinical outcome of oral cancer surgery.

The treatment modality for majority of cancer cases is surgery along with chemotherapy and/or radiotherapy. Complete removal of primary tumour with respect to precise surgical margins is an important factor deciding prognosis for many cancers including oral cancer. Despite progress in imaging techniques, the clinical outcome of the patients with oral squamous cell carcinoma (OSCC) is unsatisfactory, as the reported 5-year survival rate is 50%, which is mainly due to the incomplete removal of primary tumour^1^. The detection of oral carcinoma using 5-aminolevulinic acid (5-ALA)-induced PPIX fluorescence has recognized histologically malignant tissues and shown clear cut margins in 63% cases, keratinization of the tissues was the limitation for accurate diagnosis^2^. Many of the techniques currently used in the prediction of oral cancer margins like vital staining, fluorescent visualization and optical coherence tomography are reviewed elsewhere^3^. Two of the techniques emerging for prediction of surgical margins are radiofrequency spectroscopy and Raman spectroscopy. MarginProbe is one promising device that has come to the clinical use for the detection of surgical margins in breast cancer. The excised tissues are used for radiofrequency spectroscopy where the normal and tumour tissues are distinguished based on their dielectric properties^4^. The use of this instrument was shown to reduce the failure of margin prediction, as patients underwent surgery without MarginProbe reported positive surgical margins in 32% cases where as in the surgery aided with the device, the failure was 17%^5^. Introduction of MarginProbe reduced the re-excision rate in ductal carcinoma (DCIS) and lobular carcinomas from 61.7 to 23.1 and 37.0 to 19.0%, respectively^6^. MarginProbe is reported to reduce the re-excision rate in another clinical trial, where a total of 19.8% (59 of 298) of patients in the device arm had to go through a re-excision procedure compared with 25.8% (77 of 298) in the control arm^7^. The use of this device in other forms of cancer including oral cancer is not addressed. Further, modifications of the method are warranted to improve the success rate of disease free-margin prediction.

Recently it has been shown that surface-enhanced resonance Raman scattering (SERS) nanostars, which resonant in the near infra red (NIR) spectrum can detect macroscopic and microscopic tumour masses in genetically engineered mouse models of pancreatic cancer, breast cancer, prostate cancer, and sarcoma^8^. With this advanced technology the authors argue to detect tumour masses of size 100 μm diameter. Raman spectroscopy technique has been tested in freshly excised human tongue specimens, and the Raman spectra for tumour tissue was distinct from that of healthy adipose tissue or muscle tissue^9^. This was corroborated by another study, where OSCC spectra were shown to be distinct from the spectra of adipose tissue, nerve, muscle, gland, connective tissue, and squamous epithelium with reasonable sensitivity. But they observed that dysplastic epithelium, basal layers of epithelium, inflammation- and capillary-rich connective tissue, and connective and glandular tissue close to OSCC are difficult to discriminate from OSCC by this technique^10^. In a rat model of esophageal cancer, it was shown that the specificity of detection of tumour region is enhanced when the surface enhanced Raman scattering nanoparticle is linked to an antibody that detects EGFR or HER2. The authors showed that clear signals without nonspecificity are obtained when the detection is targeted^11^. Even though these two techniques can detect all types of cancer cells, the sensitivity and specificity of detection could be improved when combined with specific markers present in cancer cells. Molecular markers present in wide variety of cancer types will be the ideal molecules for targeting.

Gastrin releasing peptide is a regulatory peptide that acts through its receptor GRPR to regulate physiological functions in various organs. Bombesin, Gastrin Releasing peptide and neuromedin B are the ligands for GRPR, which is a G-protein receptor. The activation of this pathway can elicit various effects like cell proliferation and growth, and over-expression of GRPR is reported in a variety of cancers including head and neck cancer (HNSCC)^12^. Variants of binding peptides for GRPR are widely tested to use as a radiopharmaceutical, and for imaging of prostate cancer^13^, breast cancer and lung cancer^14^. A[68Ga]-labeled bombesin analog BAY86-7548 has been clinically tested for detection of prostate cancer^15^. The potential use of GRPR-binding peptides in the prediction of surgical margin and metastatic lymph nodes was not addressed so far. The conventional imaging techniques used in preclinical studies never revealed a metastatic lymph node and precise surgical margin in live animals^16^,^17^,^18^. But radiolabelled peptides were able to detect metastatic lymph nodes in patients^15^,^19^. Here we modified the method by incorporating a near infrared (NIR) dye conjugate to the GRPR-targeting peptide, which might be useful in intraoperative surgical margin decision as well as detecting the metastatic lymph nodes. In the present study, we used a 3D imaging technique to calculate the size of the tumour, predict the margin and metastatic lymph nodes.

## Results

### Design and synthesis of peptide, conjugation with tags and confirmation of specificity

**Figure 1:**
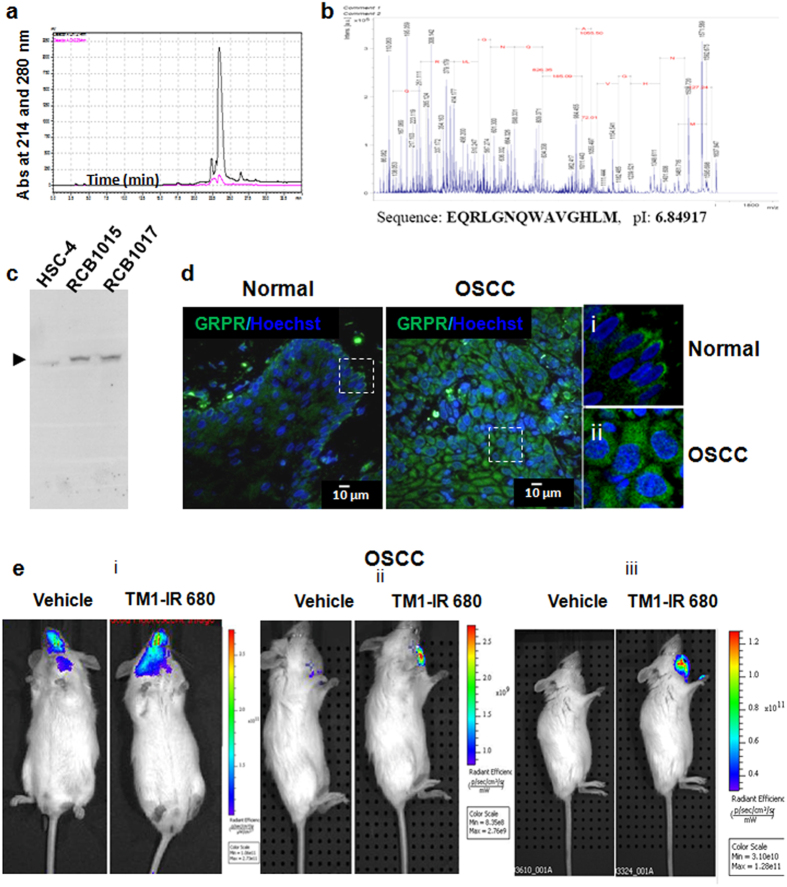
Synthesis and specific detection by TM1 peptide. **(a)** The RP-HPLC profile of the synthesized peptide. The TM1 peptide was eluted at 23.5 minutes. **(b)** The MALDI/TOF MS spectrum, sequence and isoelectic point of TM1 are shown. **(c)** The western blot analysis for testing the specificity of the peptide-targeting using biotin-tagged peptides in three oral cancer cell lines. The molecular weight of GRPR was confirmed as 90 KDa. **(d)** The cryosection from the OSCC tissue and surrounding histopathologically non malignant region was probed for GRPR using TM1-FITC. The magnified region of the white square is shown as i or ii. **(e)** Orthotopic primary OSCC tumour bearing mice were injected with TM1-IR680/vehicle (PBS), and after 43 h *in vivo* imaging was done.

A peptide that can bind to GRPR, TM1 was designed based on the sequence of the interacting protein, bombesin. The peptide was chemically synthesiszed and the purity was analyzed by HPLC using C18 reverse-phase column (Fig. 1a) and the sequence was confirmed by peptide sequencing (Fig. 1b). Biotin, FITC or IR680RD were coupled to the peptide and the conjugated peptide was purified by size exclusion chromatography. The biotin-conjugated peptide was used for western blot as described under methods. A single band of expected molecular weight was observed, showing the specificity of detection by the peptide (Fig. 1c).

### TM1-peptide for detection of oral carcinoma

When we analysed the expression of GRPR using TM1-FITC, a strong expression of GRPR was observed uniformly in primary OSCC sections as compared to respective adjacent non-malignant region of the patient, where the expression was limited to basal layer only (Fig. 1d). We have analysed the GRPR expression in nine OSCC samples along with the non-malignant region (Supplementary Table S1, Supplementary Fig. S1). The results show that the expression of GRPR in non-malignant region is drastically low, compared to OSCC with respect to level as well as extent of expression. For *in vivo* studies, an orthotopic model of oral carcinoma was used. The time of imaging was standardized using tumours formed by oral cancer cell line (Supplementary Fig. S2). The specificity of TM1 tagged with IR680RD was confirmed by *ex vivo* imaging of organs and confocal microscopy of live tumour sections (Supplementary Fig. S2). The efficiency of detection by TM1-IR680 was comparable to bioluminescence when oral cancer cells expressing luciferase were used at different cell numbers to make the orthotopic model and imaged at different time points (Supplementary Fig. S3). Keratinocytes isolated from primary OSCC samples were used to make orthotopic models, and TM1-IR680 was able to detect tumour in all the three samples (Fig. 1e).

### 3D imaging to predict the tumour size and metastatic lymph nodes

**Figure 2:**
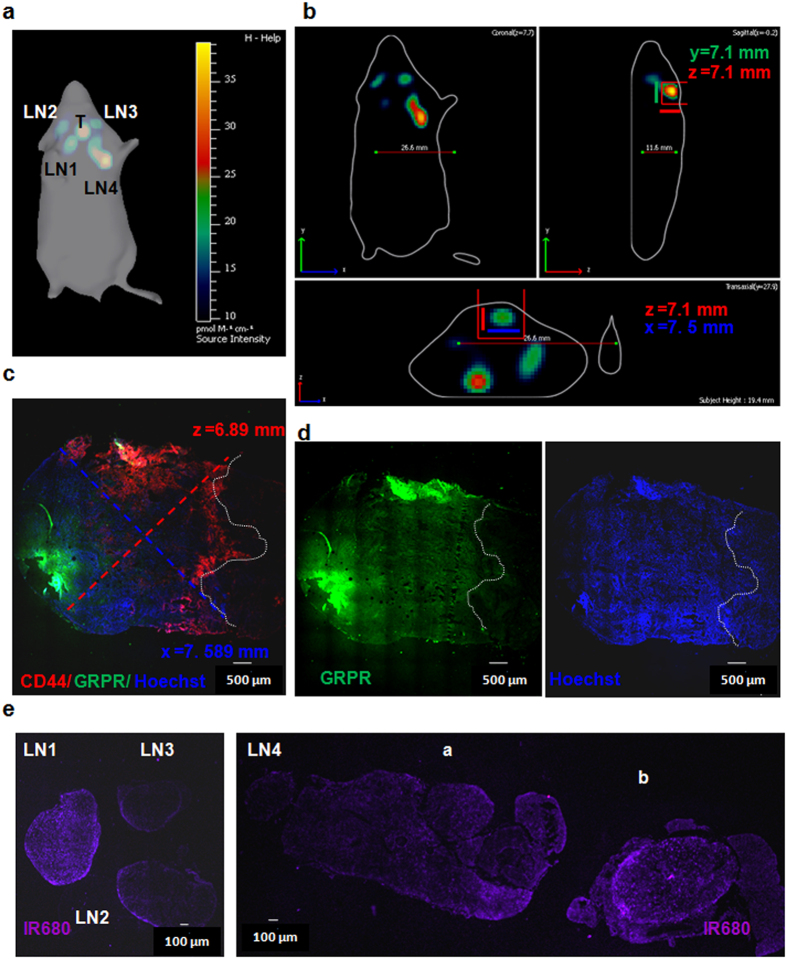
3D imaging using TM1-IR680. **(a)** The 3D image was acquired. The tumour was selected as shown in the red box. The dimensions were measured using the software Living Image. **(b)** The tumour sections were probed with TM1-FITC and CD44-PE. The image was acquired with image stitching and Z-stacks. The extent of invasion was demarcated by GRPR and CD44 expression in a suitable plane giving maximum width. The length was measured using NIS Element software. **(c,d)** The expression of GRPR and CD44 in the merged Z-stack is shown. Hoechst staining shows the nuclei. The dotted line represents the tumour margin. **(e)** Live Cryosections of lymph nodes (LN1,2,3 and 4) were imaged for IR680 using confocal microscope.

Figure 2A represents the 3D imaging done for one of the samples (as shown in Fig. 1e iii) using FLIT mode to visualise the localization of the TM1-IR680 on a 3D projection of the animal. We detected primary tumour and 4 localized regions corresponding to superficial cervical lymph nodes (LN2 and LN3), right deep cervical (LN1), and a fused structure of left deep cervical and brachial nodes (LN4) (Fig. 2a). The enlargements of all the lymph nodes except LN3 were prominent. The size of the tumour was measured using the software as 7.5 mm × 7.1 mm × 7.1 mm (Fig. 2b). Since this is the orthotopic tumour, the tumour infiltrates to underlying tissues. So a clear demarcation of the tumour margin is relevant as in human surgery. We excised the tumour including the surrounding normal tissue and live sections were taken with minimum exposure to light. Since there was a partial quenching in the fluorescence of IR680 in the sections, which may affect the measurements of tumour dimensions, we stained the live sections with CD44-PE and TM1-FITC. Hoechst 33342 was added to visualize nuclei. With the help of image stitching tool of the software the dimensions were taken. The white line in Fig. 2c indicates the margin drawn with TM1-FITC. Same margin was overlaid for all the channels. The CD44 PE and TM1-FITC showed co-localization, and Hoechst revealed non-epithelial tissue beyond the margin (Fig. 2d). X and Z axes were marked according to the orientation to the body wall. The measurements for tumour region of the section were 7.589 mm × 6.89 mm (Fig. 2c).

### *In vivo* imaging by TM1-IR680 is sensitive to detect dispersed tumour cells, which are not colonized, in lymph nodes

**Figure 3:**
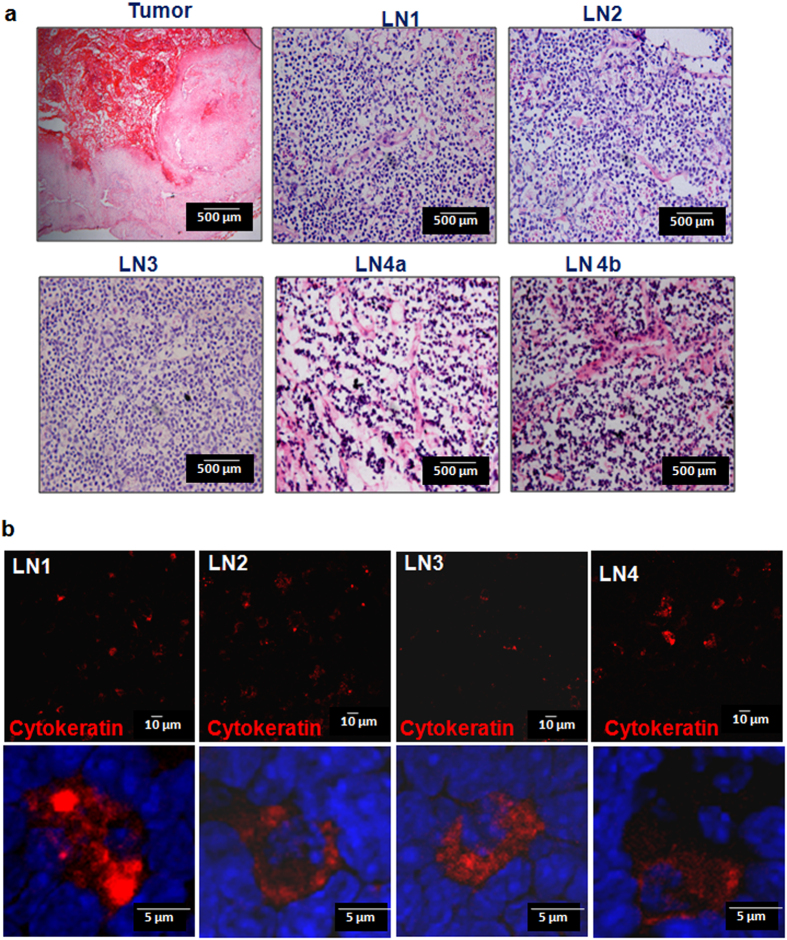
Confirmation of tumour cells in primary tumour and lymph nodes. **(a)** The cryosections were fixed with 4% PFA and then stained with haematoxylin/eosin, and images were acquired. (**b**) The lymph node sections were stained for cytokeratin 14 and 18. The lower panel shows the higher magnification of the same.

The live tissue sections of all the lymph nodes were imaged for IR680 in a confocal microscope. As shown in Fig. 2e, the fluorescent intensity for IR680 in LN2 and LN3 were low, may be due to the quenching of the fluorophore during the processing. Since the detection of metastatic tumour cells in the lymph nodes was difficult, serial sections were stained after a brief fixation, for GRPR and CD44 (an epithelial marker). The specificity of detection on cryosections by TM1-FITC was confirmed (Supplementary Fig. S4). GRPR^+^ CD44^+^ cells in a group of two or three were scattered in the sections confirming the presence of tumour cells (Supplementary Fig. S5). Conventional Haematoxylin/eosin staining was done on sections after fixation of the tissue (Fig. 3). A pathologist, however, graded the nodes as reactive nodes without tumour cells as the single cells scattered was not visible under histopathological examination (Fig. 3a). To confirm the presence of tumour cells, Cytokeratin staining was also done on fixed sections, which also revealed metastatic cells, which were scattered (Fig. 3b). A co-staining for CD44 and Von Willebrand Factor, a marker for endothelial cells, platelets and megakaryocytes showed that the CD44^+^ cells are distinct from the cells expressing Von Willebrand Factor (Supplementary Fig. S5).

### TM1-IR680 is safe for *in vivo* administration

**Figure 4:**
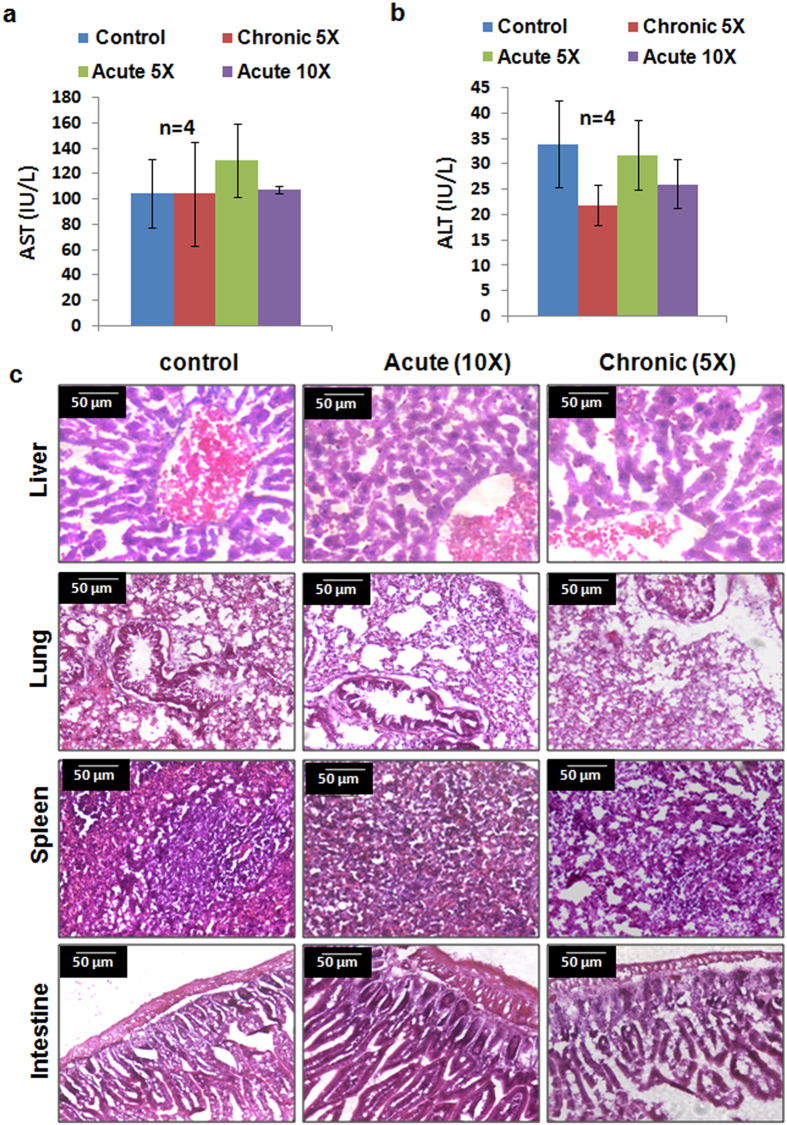
Toxicity analysis of TM1 administration. **(a,b)** The peptide administration for the respective group was done as described under methods section and the serum ALT and AST levels are shown in the graph. N represents the number of animals. Error bar represents standard deviation (**c**) Haematoxylin/eosin staining of different organs collected from respective groups are shown.

To ascertain whether the peptide is cleared from the body after the administration, and to study the immune response and/or toxicity of the peptide, studies were conducted in Swiss albino mice, which is an immunocompetent mouse and a better model for these studies than immunocompromised NOD/SCID. TM1 peptide was cleared from circulation within 4 h and from the tumour by 72 h (Supplementary Fig. S6). We have monitored the animals for one month after the administration of the peptide for any visible change in food uptake, behaviour or loss of weight, and no changes were observed. The toxicity was further assessed by acute (5 × and 10 × ) and Chronic (5 × ) treatment as described under Methods section. Serum Aspartate Aminotransferase (AST) and Alanine Aminotransferase (ALT) levels did not show any significant variation (Fig. 4a,b). Histopathological analysis of different organs showed no significant changes in the treated group compared to control (Fig. 4c). Moreover, treatment of the peptide up to 48 h did not elicit any significant change in tumour properties like, proliferation or EMT (Supplementary Fig. S6).

## Discussion

Even though MarginProbe and SERS could detect tumour areas based on the physical properties of malignant cells, current situation demand technologies to improve their sensitivity and specificity. Since it has been shown that coupling this with targeted imaging tools could improve the specificity^11^, we need to have probes that target cancer specific molecules and technologies that are suitable for the type of cancer. Optical imaging is a relatively inexpensive, fast, and sensitive imaging approach for the non-invasive detection of human cancers in locations that are accessible by an optical imaging device. Fluorescent-based optical imaging using Near Infrared (NIR) dyes has emerged as an optimal tool for this, as they offer low background and better penetration capability. Optical imaging using NIR fluorophores are feasible for cancers that are not deeply located, like breast cancer, colorectal cancer and oral cancer^20^. The fluorophores currently used in image-guided surgery for sentinel lymph node mapping and tumour mapping are indocyanine green (ICG) and 5-ALA^20^. As these two agents are not ligand-targeted contrast agents, considerable efforts were taken to develop targeted probes that could be used for image-guided surgery. Recently it has been shown that an NIR dye linked anti-EGFR molecule (cetuximab-IRDye800CW), a drug given for treatment can be used to visualize the localization of the molecule in human patients^21^. Even though they showed the efficacy of the probe to detect primary tumours how far this could detect dispersed tumour cells and metastatic regions were not studied.

It is reported that GRPR over-expression is an early event of OSCC progression, and the adjacent non-malignant region have higher amount of the molecule than the epithelium of healthy individuals^22^,^23^, which raised a question whether it will be a suitable molecule for margin prediction in OSCC. These two studies have also revealed that the up-regulation of GRPR in OSCC is considerably higher than the adjacent non-malignant region which was confirmed by our immunohistochemical analysis. Since GRPR is over-expressed in a wide variety of cancers, bombesin analogue peptides were used extensively for cancer imaging, for detection of cancer. Yet, its potential for intraoperative surgical margin assessment or metastatic lymph node was not addressed, primarily due to the limitation of imaging resolution obtained in the preclinical studies. Many of the studies were conducted in xenografts, where the surgical margin and lymph node metastases are not relevant. In one of the studies that claimed to detect metastatic lymph nodes in prostate cancer, the metastatic lymph nodes were detected only after removing the internal organs, not in live animal^13^, which might be due to the limitation of imaging modality used. One of the factors that could affect the specificity of detection is the specificity of the peptide in target detection. We have shown that it interacts only with GRPR by western blot, a modified technique with biotin labelled peptide, which we have published recently^24^. Further, TM1-IR680-based 3D imaging can detect primary cancer and metastatic lymph nodes, which was not possible by conventional *in vivo* imaging. The important aspect we have shown, for the first time, is that we can predict the three dimensional margins of the tumour based on GRPR expression, which is corroborated by CD44 staining. Even though we show the margin prediction using TM1-IR680, there are certain factors that decide the accuracy of detection by NIR imaging. The composition and thickness of the skin, the amount of hemaglobin, the sensitivity of the camera etc. can influence the sensitivity of detection^25^.

Even the most sensitive imaging techniques currently used detects micrometastases greater than 100 μm diameter^8^,^9^,^13^,^20^,^26^. However, using our peptide probe, the lymph nodes that had only dispersed tumour cells, which would have identified as negative nodes by conventional histopathological examination, were also detected. Lymph nodes which harbour dispersed tumour cells might show node positivity later, after colonization of the dispersed tumour cells. Thus removal of those nodes is important in the prognosis. Here we show that TM1-IR680 could detect those nodes, which could improve the surgical outcome. Moreover, the same peptide can also be used to confirm the margin negativity during surgery on excised tissues, since the protocol for staining the sections with peptide is specific and requires less than one hour as shown in Fig. S3 and methods sections. In conclusion, we report a specific peptide probe for the detection of tumour cells with high sensitivity. The techniques employed reveal its potential to be used as a probe for intraoperative surgical margin assessment.

## Methods

### Synthesis of peptide

C-terminally amidated peptide (TM1) was synthesized by manual solid phase peptide synthesis technique using CLEAR amide resin as the solid support, following the 9-fluorenylmethoxy carbonyl (Fmoc) chemistry and purified by RP-HPLC using C18 column (Shimadzu, Tokyo, Japan). Homogeneous purification and mass accuracy were confirmed by semi-preparative RP-HPLC (Shimadzu, Tokyo, Japan) and MALDI TOF MS analysis (Bruker, Bremen, Germany). The peptide sequence analysis was performed on *ultrafleXtreme* MALDI TOF/TOF system (Bruker, Bremen, Germany). Labeling reaction was done by mixing 1 mg/ml of FITC (Sigma, IN, USA) or IR680RD NHS ester (LI-COR Biosciences GmbH, Germany) to 1 mM peptide solution, adjusted to pH 9.7 with Na_2_CO_3._ Biotinylation of the peptide was done using a Biotinylation kit (GeNei, Bangalore, India) following the manufacturer’s protocol.

### OSCC samples

Primary OSCC samples and adjacent non-malignant region were collected from oral cancer patients of Regional Cancer Centre, Thiruvananthapuram after getting informed consent from all the patients. All the protocols were approved by Institute Human Ethical Committee of RCC (HEC 34/2011) and RGCB (IHEC/1/2011/04). All the protocols were performed in accordance with the guidelines of IHEC of RCC and RGCB.

### Oral cancer cell lines

OSCC cell lines HSC-4, RCB1015 and RCB1017 were obtained from RIKEN BRC, Japan. The genetic identity of cell lines was last authenticated 3 months before performing experiments, through short tandem repeats (STR) analysis. The cell lines were maintained in DMEM (Invitrogen ^TM^, CA, USA) supplemented with 10% FBS (Invitrogen ^TM^, CA, USA), 1Xpenicillin/streptomycin and maintained at 37 °C in 5% CO_2_.

### Western Blotting

Western blotting was performed as described before^27^. Briefly, cell lysates were prepared in RIPA lysis buffer and the bands were resolved by 10% SDS-PAGE, and transferred to nitrocellulose membrane for immunoblot analysis. Biotin-tagged peptides were added, and the bound peptide was detected using streptavidin-HRP (Thermo Fisher Scientific, MA, USA). The blot was developed using ECL plus western blotting developing system (GE healthcare, Buckinghamshire, UK) and imaging was done using Versa Doc 4000MP (Bio-Rad, CA, USA).

### Immunohistochemistry

Cryosections (7 μm) of fixed tissues (4%PFA) were taken using Cryostat LeicaCM1850 UV (Leica Biosystems, Wetzlar, Germany), and the antigen retrieval was done with 0.01 M citrate (pH 6.0) and permeabilized with 0.3% Triton X-100/PBS (PBST) and blocked with 2% serum as described previously^28^. Primary antibody or peptide tagged with FITC was added and incubated at 4 °C, overnight. Secondary antibody was added and incubated for one hour. Hoechst 33342 (Sigma, IN, USA) was used to stain the nuclei, and antifade was used for mounting. Images were acquired using NikonA1R LSCM confocal microscope (Nikon, Tokyo, Japan). The antibodies used were against pancytokeratin (Santa Cruz Biotechnology Inc, TX, USA), CD44-PE (BD Pharmingen, CA, USA), and Von Willebrand Factor (Abcam, Cambridge, UK).

### Animal models for oral carcinoma

NOD.CB17-*Prkdcscid*/J mice (The Jackson Laboratory, BarHarbor, USA) were used to make orthotopic floor of the mouth oral cancer model^29^. All the protocols were approved by, and performed in accordance with the guidelines and regulations of with Institute Animal Ethical Committee (IAEC) of RGCB. Oral cancer cell line, HSC-4, (2 × 10^6^ cells/mice) was injected with Corning Matrigel growth factor reduced (CORNING, NY, USA) to the floor of the mouth region superficial to the mylohyoid muscle, for the orthotopic model. Primary OSCC cells (50000) were grown as tumour spheres grown in suspension using Opti-MEM (1 × ) Reduced serum (Invitrogen ^TM^, CA, USA) medium supplemented with N/2, 20 μg/ml each of EGF and bFGF (Invitrogen ^TM^, CA, USA), and 1 × Insulin-Transferrin-Selenium (Invitrogen ^TM^, CA, USA) for 6 days, and the spheres were injected to make orthotopic model as described earlier.

### *In vivo* imaging

Mice bearing tumour models were imaged using IVIS SPECTRUM *in vivo* imaging system (Perkin Elmer, CA, USA) under 3% isofluorane anesthesia. For fluorescent acquisition, TM1-IR680 peptide was injected through tail vein (160 pmols/Kg bodyweight) 42 h before, unless otherwise mentioned. The background subtraction was based on the fluorescence intensity on muscle. 3D imaging was done by fluorescence imaging tomography (FLIT). The calculations were done using IVIS SPECTRUM imaging software Living Image, Version 4.3.1.

### Staining with TM1-FITC on cryosections

Live tissues were collected, blood was washed off and excess PBS was briefly dried over a tissue paper. Tissues were mounted in OCT and 5 μm sections were taken. The slides were immersed in PBS for 5 minutes and fixed with 4% PFA for 7 minutes. The sections were washed with PBS and TM1-FITC was incubated for 30 minutes. Excess peptides were washed off and the sections were visualized under microscope.

### Toxicity analysis

Toxicity analysis of the peptide was performed in Swiss albino mice (Piramal Enterprise Limited, Mumbai, India) All the protocols were approved by, and performed in accordance with the guidelines and regulations of IAEC of RGCB. For acute treatment, 10- and 5-folds higher doses of the required concentration was administered in a single dose; and 48 h later, the blood was collected. For chronic treatment, 5-fold dose was administered for 20 days on alternate days to get a total of 10 doses. 48 h after the administration of last dose, the blood was collected. Blood samples were collected by cardiac puncture and serum was separated. Serum ALT and AST activity were measured using a commercial kit from AGAPPE Diagnostics (Chennai, India) following the manufacturer’s protocol.

### Histopathological analysis

After the peptide treatment, organs were collected, fixed in 4% PFA and cryosections were taken. Haematoxylin/eosin staining was performed following the standard protocol. For the live sections of tumour and lymph nodes collected after 3D imaging, we fixed the sections in 4% PFA for 10 minutes, and proceeded with the rest of the steps.

## Additional Information

**How to cite this article**: Suganya S. *et al.* TM1-IR680 peptide for assessment of surgical margin and lymph node metastasis in murine orthotopic model of oral cancer. *Sci. Rep.* **6**, 36726; doi: 10.1038/srep36726 (2016).

**Publisher’s note**: Springer Nature remains neutral with regard to jurisdictional claims in published maps and institutional affiliations.

## Supplementary Material

Supplementary Information
