# Supplementary material for: TM1-IR680 peptide for assessment of surgical margin and lymph node metastasis in murine orthotopic model of oral cancer
Source: Sci Rep. 2016 Nov 9;6:36726. doi: 10.1038/srep36726 (PMC5101486; doi:10.1038/srep36726)
Supplement: Supplementary Information [file srep36726-s1.pdf]

TM1-IR680 peptide for assessment of surgical margin and lymph node metastasis in murine orthotopic model of oral cancer

Annie A. Suganya S. [a], Kochurani, K.J[a]., Madhumathy .G. Nair[a], Jiss Maria Louis[a], Santhosh Sankaran[b], Rajagopal R[b]., Santhosh Kumar K.[c], Parvin Abraham[c], Balagopal P.G[d], Paul Sebastian[d], Thara Somananthan[e] and Tessy Thomas Maliekal[a*]

List of Supplementary materials

Supplementary Table S1

Figure S1 Comparison of GRPR expression in non malignant and malignant regions from oral cancer patients using TM1-FITC.

Figure S2 *In vivo* detection of oral cancer using TM1-IR680.

Figure S3 Comparison of detection using fluorescence and bioluminescence.

Figure S4 Detection by TM1-FITC on live tissue.

Figure S5 Confirmation of lymph node metastasis.

Figure S6Safety aspects of TM1 administration.

Supplementary Methods

Supplementary Figure legends

Sample	Normal		OSCC	
	Expression level	% of basal layer cells	Expression level	% of tumor cells
TM20	Mild	22	Intense	92
TM21	Moderate	25	Moderate	53
TM22	Mild	64	Intense	81
TM23	Mild	21	Intense	91
TM24	Mild	35	Intense	45
TM25	Mild	32	Moderate	44
TM26	Mild	30	Intense	79
TM27	Mild	34	Intense	82
TM28	Moderate	40	Intense	80

Supplementary Table S1 Expression analysis of GRPR in malignant tissue and adjacent non-malignant tissue. The malignant (OSCC) and adjacent non-malignant (normal) region from each patient was collected. Sections were stained with TM1-FITC. 4 sections from each patient were scored for the expression. The average expression levels of the 4 sections were grouped as mild, moderate or intense. Two representative pictures (60×) from each section were taken and the % of cells expressing GRPR was calculated from the cumulative number from the patient.

Figure S1

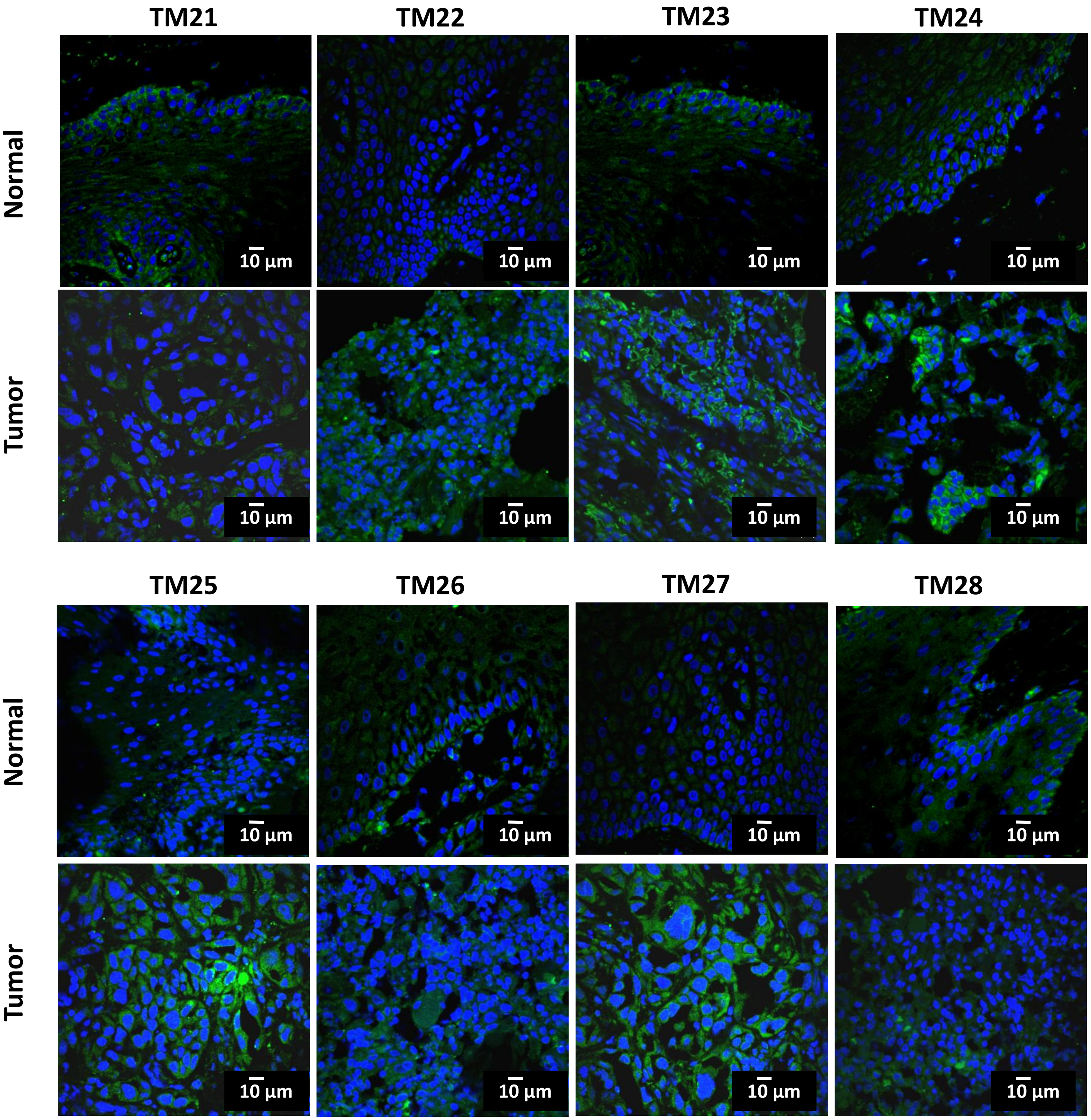

TM21
TM22
TM23
TM24
Normal
Tumor
TM25
TM26
TM27
TM28
Normal
Tumor
10 µm

Figure S2

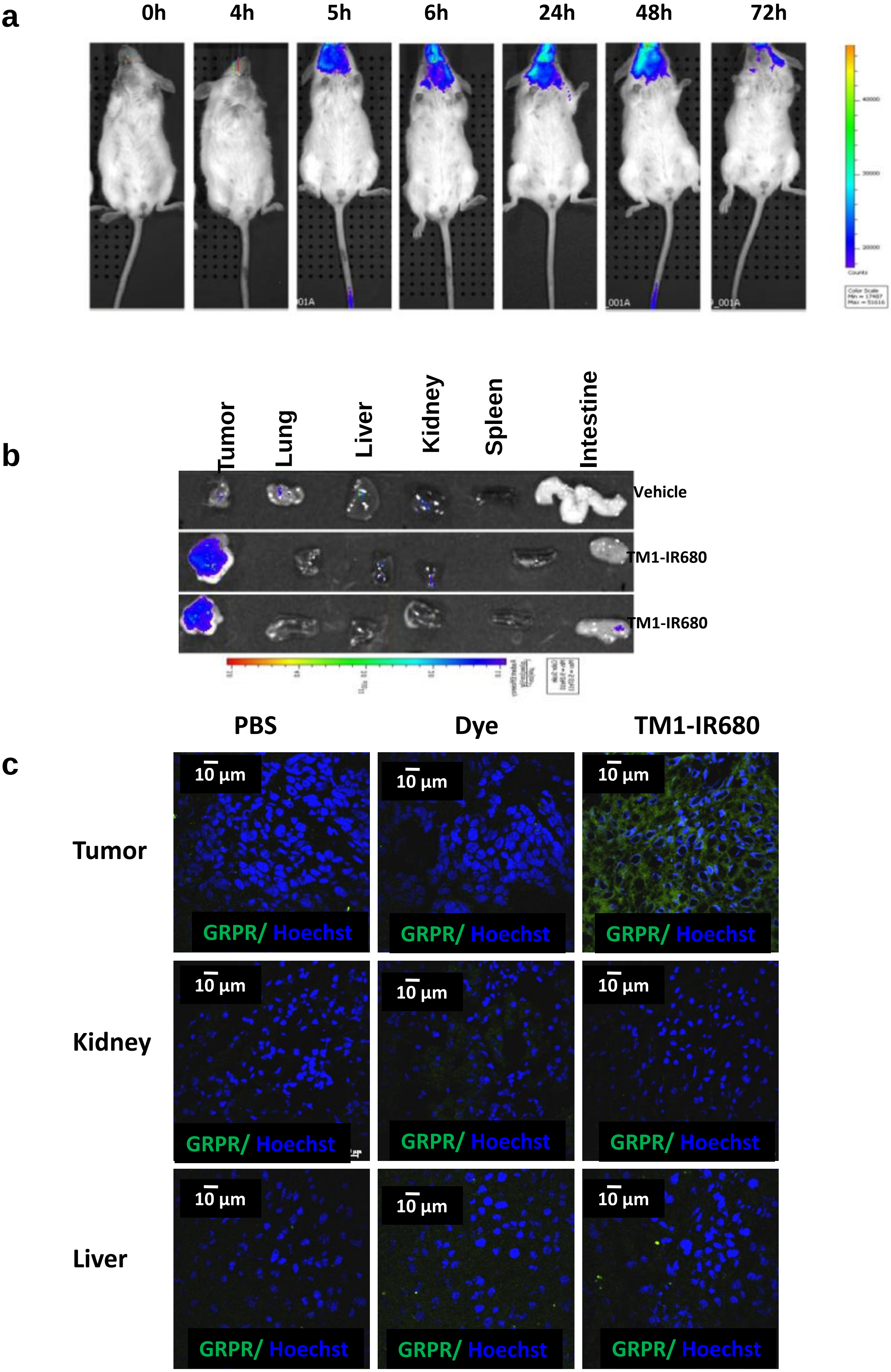
a
0h
4h
5h
6h
24h
48h
72h
b
Tumor
Lung
Liver
Kidney
Spleen
Intestine
Vehicle
TM1-IR680
TM1-IR680
c
PBS
Dye
TM1-IR680
Tumor
Kidney
Liver
10 μm
GRPR/ Hoechst

Figure S3

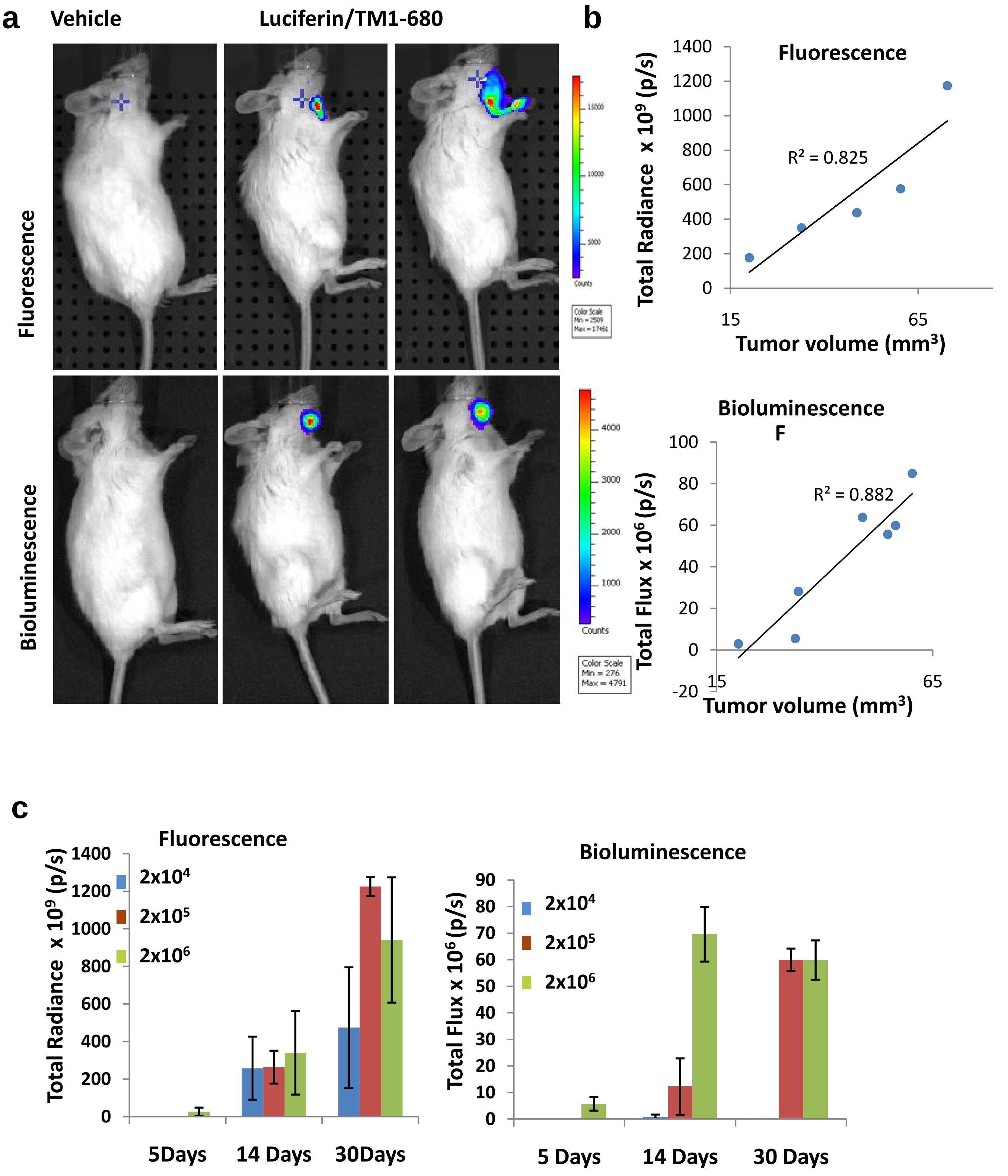
a
Vehicle
Luciferin/TM1-680
Fluorescence
Bioluminescence
Counts
Color Scale
Min = 2509
Max = 17461
Color Scale
Min = 276
Max = 4791
b
Fluorescence
Total Radiance x 10⁹ (p/s)
R² = 0.825
Tumor volume (mm³)
Bioluminescence
F
Total Flux x 10⁶ (p/s)
R² = 0.882
Tumor volume (mm³)
c
Fluorescence
Total Radiance x 10⁹ (p/s)
2x10⁴
2x10⁵
2x10⁶
5Days
14 Days
30Days
Bioluminescence
Total Flux x 10⁶ (p/s)
2x10⁴
2x10⁵
2x10⁶
5 Days
14 Days
30 Days

Figure S4

live tissue

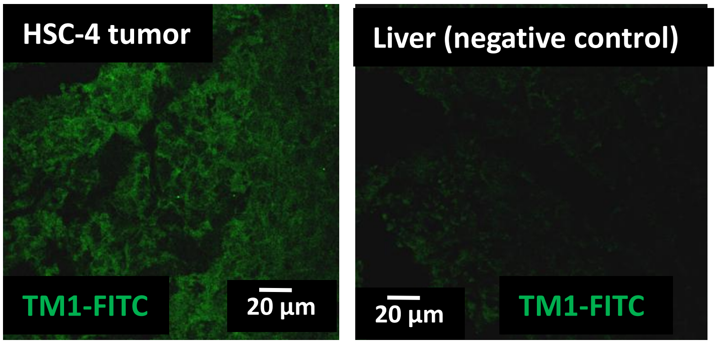

Figure S5

a

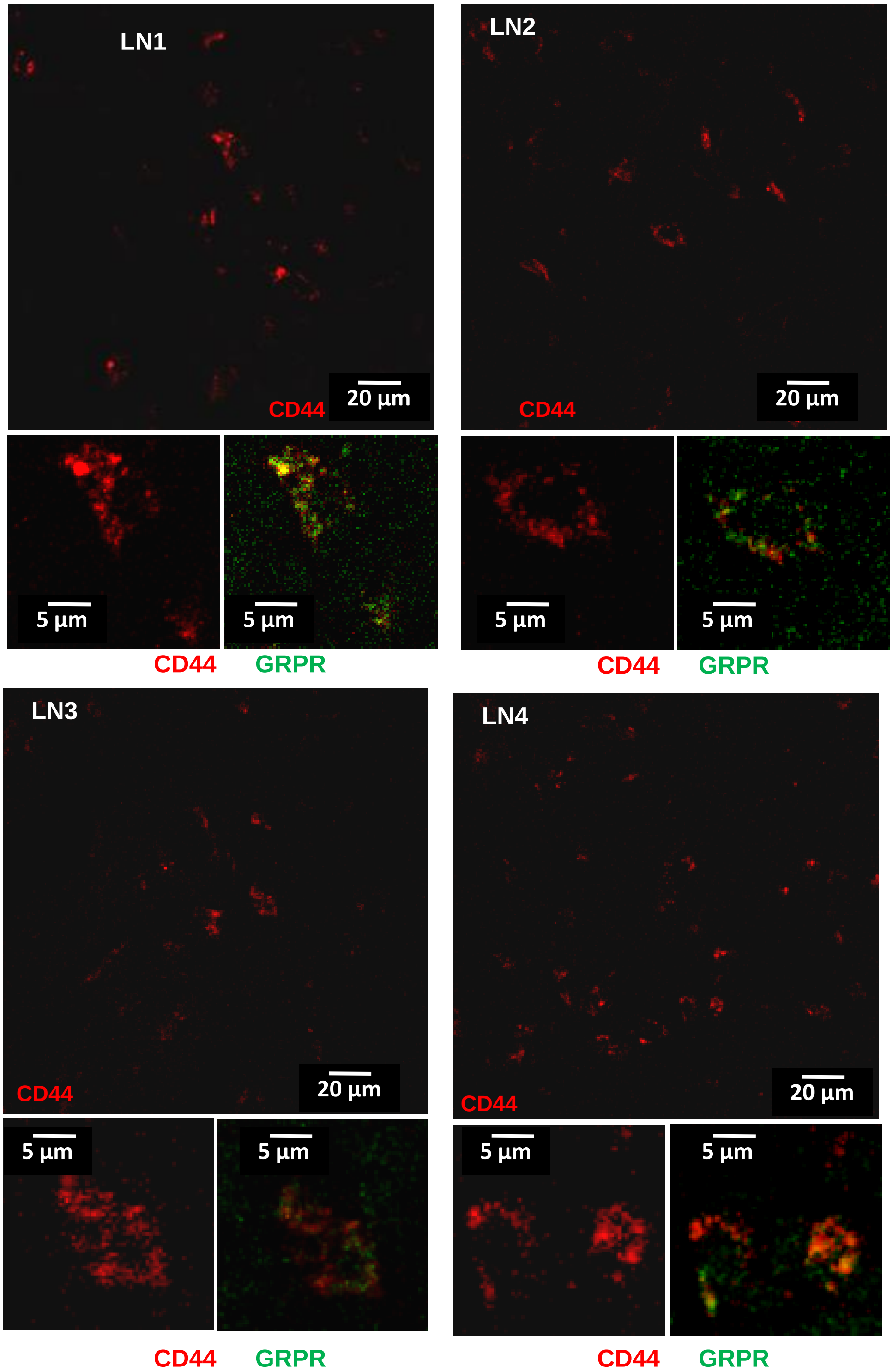
LN1
CD44
20 µm
LN2
CD44
20 µm
5 µm
5 µm
5 µm
5 µm
CD44 GRPR
CD44 GRPR
LN3
CD44
20 µm
LN4
CD44
20 µm
5 µm
5 µm
5 µm
5 µm
CD44 GRPR
CD44 GRPR

b

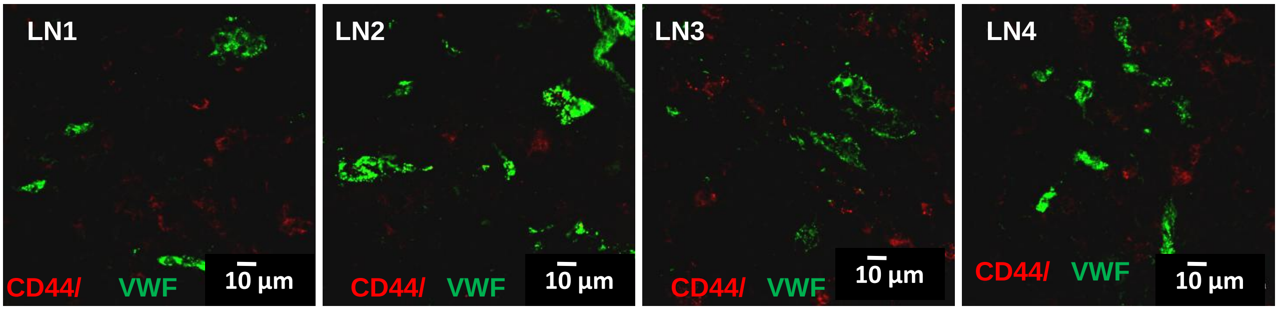
LN1
CD44/ VWF
10 µm
LN2
CD44/ VWF
10 µm
LN3
CD44/ VWF
10 µm
LN4
CD44/ VWF
10 µm

Figure S6

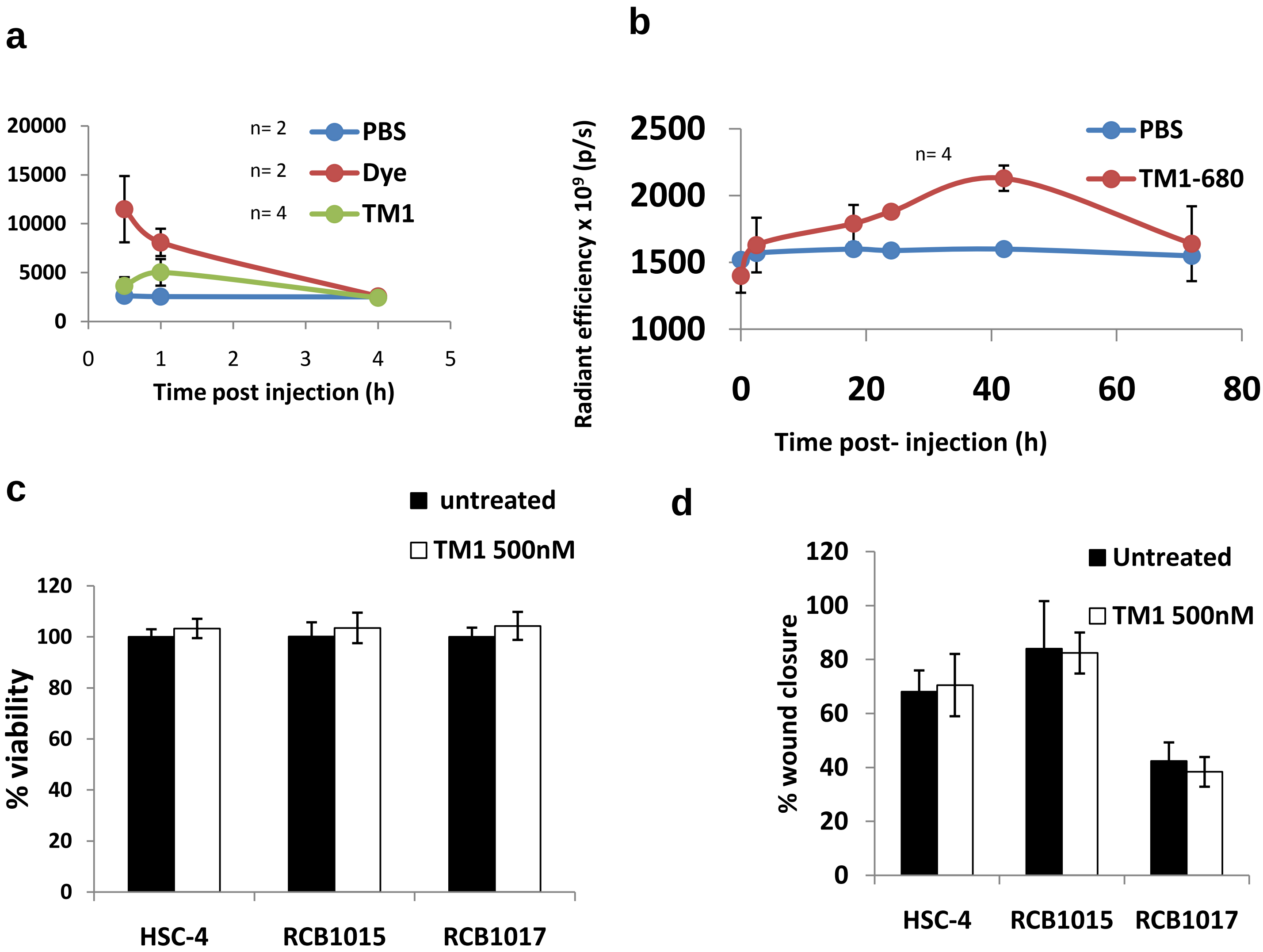
a
b
c
d
n= 2
n= 2
n= 4
PBS
Dye
TM1
20000
15000
10000
5000
0
0
1
2
3
4
5
Time post injection (h)
Radiant efficiency x 10⁹ (p/s)
2500
2000
1500
1000
n= 4
PBS
TM1-680
0
20
40
60
80
Time post- injection (h)
untreated
TM1 500nM
% viability
120
100
80
60
40
20
0
HSC-4
RCB1015
RCB1017
Untreated
TM1 500nM
% wound closure
120
100
80
60
40
20
0
HSC-4
RCB1015
RCB1017

Supplementary methods

Fluorimetric analysis of serum samples

Swiss albino mice were injected with the IR680-labelled peptide as described and serum samples were collected by submandibular bleeding. Serum was separated and the fluorescence was measured (Ex 672/9 and Em 700/20) using infinite M200 fluorimeter (TECAN, Männedorf, Switzerland).

Bioluminescence imaging

NOD.CB17-*Prkdcscid*/J mice bearing orthotopic tumours were used for *in vivo* imaging. For comparison of bioluminescence and fluorescence, we generated luciferase expressing HSC-4 cells by stably transfecting pGL4.51(luc2/CMV/Neo) vector (Promega, WI, USA), and those cells were used for making orthotopic model. For bioluminescence imaging, the substrate D-luciferin (Promega, WI, USA) was injected intraperitoinally (80mg/ Kg body weight) and imaging was done after 14 minutes.

Tumour volume measurement

The tumour volume measurement using a digital Vernier caliper was started when the tumour was palpable, and the tumour volume was calculated as width^2 × length/2.

MTT Assay

For MTT assay, the peptide was washed off and MTT (1mg/ml) (Sigma, IN, USA) was added for 4hours, and the assay was performed as reported previously [26].

Trypan blue assay

After the treatment the cells were trypsinized, mixed with trypan blue (1:1) (Sigma, IN, USA)

and the number of viable cells and dead cells (blue) were counted to represent the % viability.

Wound healing assay

After the treatment, wound was made with pipette tip and the wound closure was imaged at the represented time intervals using Olympus IX 71 microscope (Olympus, Tokyo, Japan) and the percentage of closure was calculated using Image J software.

Supplementary figure legends

Figure S1 Comparison of GRPR expression in non malignant and malignant regions from oral cancer patients using TM1-FITC. The malignant (OSCC) and adjacent non-malignant (normal) region from each patient was collected. Sections were stained with TM1-FITC. Images were acquired using confocal microscope.

Figure S2 *In vivo* detection of oral cancer using TM1-IR680. (a) Fluorescence captured from orthotopic tumour bearing mouse injected with TM1-IR680 at different time intervals post-injection using IVIS spectrum *in vivo* imager. **(b)** Detection of fluorescence in organ of TM1-IR680/ vehicle (PBS) injected mice after 48h of peptide administration. (c) Cryosections of the live tissues of tumour, liver or kidney from NOD.CB17-*Prkdcscid*/J mice injected with PBS, unconjugated dye or TM1-IR680 were collected 24h post-injection and fluorescence of IR680 was captured using confocal microscope.

Figure S3 Comparison of detection using fluorescence and bioluminescence. (a) Orthotopic tumour bearing mouse was injected either with TM1-IR680 or PBS, and imaged after 48h for fluorescence, and then the animal received saline or luciferin substrate as intraperitonial and imaged for bioluminescence**.**

(b) Graph shows the correlation of tumour volume (measurable tumour) with respect to its total radiance/total flux in case of fluorescence and bioluminescence respectively. **(c)** Graph represents orthotopic tumour bearing mouse with different cell number and its respective fluorescence/bioluminescence emission compared at different days interval.

Figure S4 Detection by TM1-FITC on live tissue. The tumour from HSC-4 xenograft, or mouse liver tissue was stained as described under methods section.

Figure S5 Confirmation of lymph node metastasis. (**a**) Cryosections of lymph nodes were probed using TM1-FITC and CD44-PE. **(b)** Lymph node sections were co-stained for CD44 and Von Willebrand Factor.

Figure S6 Safety aspects of TM1 administration. (**a**) Shows the rate of clearance of TM1-IR680/PBS/dye from the serum. The fluorescence was measured (Ex 672/9 and Em 700/20) using fluorimeter. **(b)** Graph represents the gradual decrease in fluorescence intensity in TM1-IR680 injected orthotopic tumour bearing mouse compared with PBS injected control group.Cryosections of lymph nodes were probed using TM1-FITC and CD44-PE. (**c**) Shows the percentage viability of oral cancer cell line after 48h peptide treatment in comparison with untreated cells. (**d**) Shows the percentage of wound closure in oral cancer cell lines after 48h with or without peptide treatment.
